# Shaft Diameter Measurement Using Structured Light Vision

**DOI:** 10.3390/s150819750

**Published:** 2015-08-12

**Authors:** Siyuan Liu, Qingchang Tan, Yachao Zhang

**Affiliations:** College of Mechanical Science and Engineering, Jilin University, Changchun 130022, China; E-Mails: siyuan12@mails.jlu.edu.cn (S.L.); zhangyc14@mails.jlu.edu.cn (Y.Z.)

**Keywords:** image measurement, shaft diameter, structured light vision

## Abstract

A method for measuring shaft diameters is presented using structured light vision measurement. After calibrating a model of the structured light measurement, a virtual plane is established perpendicular to the measured shaft axis and the image of the light stripe on the shaft is projected to the virtual plane. On the virtual plane, the center of the measured shaft is determined by fitting the projected image under the geometrical constraints of the light stripe, and the shaft diameter is measured by the determined center and the projected image. Experiments evaluated the measuring accuracy of the method and the effects of some factors on the measurement are analyzed.

## 1. Introduction

Shafts are one of most important machine elements and the accuracy of machined shafts has a significant effect on the properties of any mechanical device that employs one. The accuracy of a machined shaft can be ensured using a Computer Numerical Control lathe, but mechanical wear in a CNC lathe system, system deformation, and the radial wear of the machining tools will reduce the accuracy of the machined shaft. If the variation of the shaft diameter could be monitored during its machining, the dimensional accuracy of the product could be ensured by the automatic compensation system of the CNC lathe. Therefore, on-line measurement of shaft size is very important in machining. 

**Figure 1:**
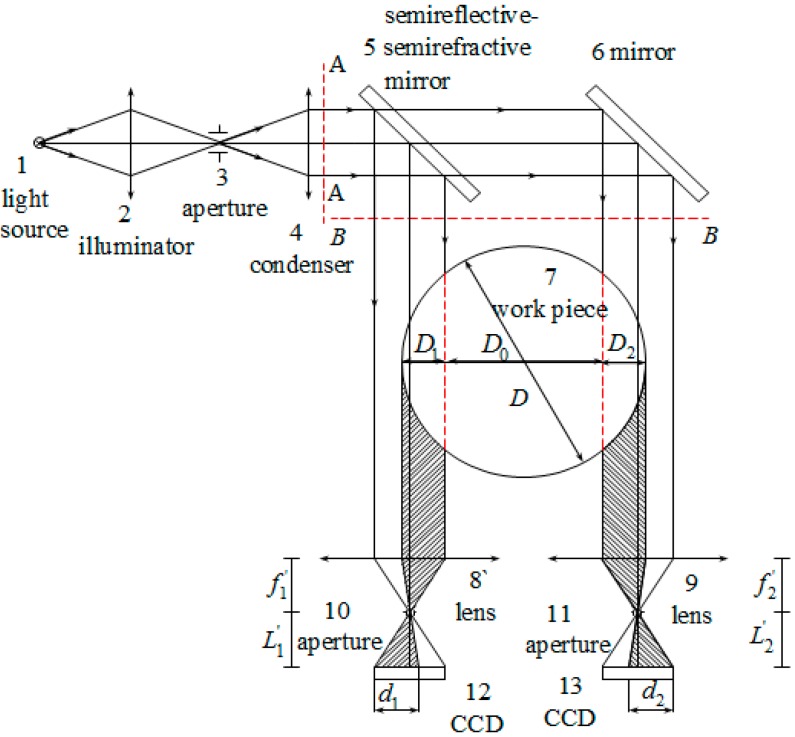
The optical principle of the parallel light projection method with double light paths (with permission from [8]).

A variety of noncontact machine vision methods for measuring the product diameters have been proposed [1,2,3,4,5]. These methods can be classified as active methods and passive methods. Passive measurement methods use a camera or two cameras to obtain shaft diameters [6,7]. In 2008, Song *et al.* [8] proposed a method where both edges of a shaft were imaged onto two cameras through two parallel light paths as shown in Figure 1 [8]. Using this approach, the shaft diameter was obtained by measuring the length of the photosensitive units on the shadow field of the two CCDs (Charge-Coupled Device) and the distance between the two parallel light paths. This method can amplify the measurement range while ensuring the measuring accuracy; however, it is very difficult to calibrate the position of the two cameras. 

**Figure 2:**
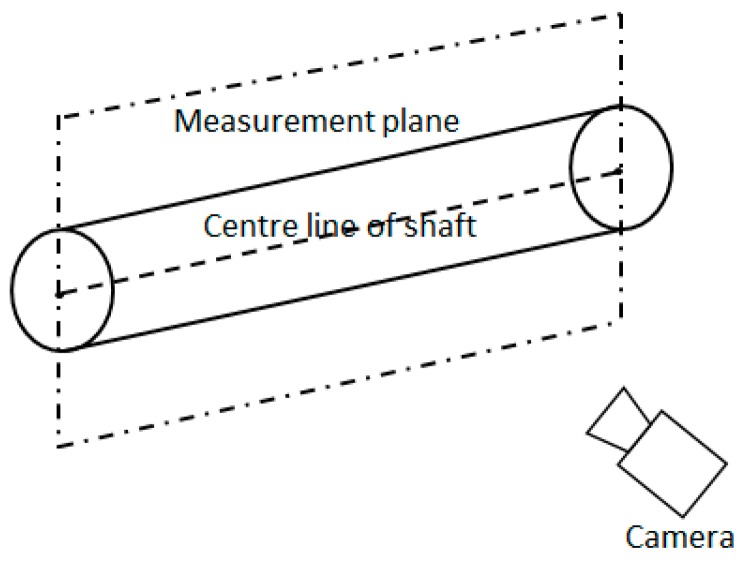
The orientations of the shaft and the measurement plane in 3D space (with permission from [9]).

In 2013, Sun [9] used a camera to obtain shaft diameters, employing a theory of plane geometry in the cross-section, which is perpendicular to the axis of the shaft, as shown in Figure 2 [9]. This method can achieve a high measuring accuracy, but it requires the calibration of the plane of the center line of the shaft before the measurement. In practice, the position of the center line changes each time the shaft is clamped by the jaw chucks of the lathe. This change of position will decrease the accuracy of measurement. 

**Figure 3:**
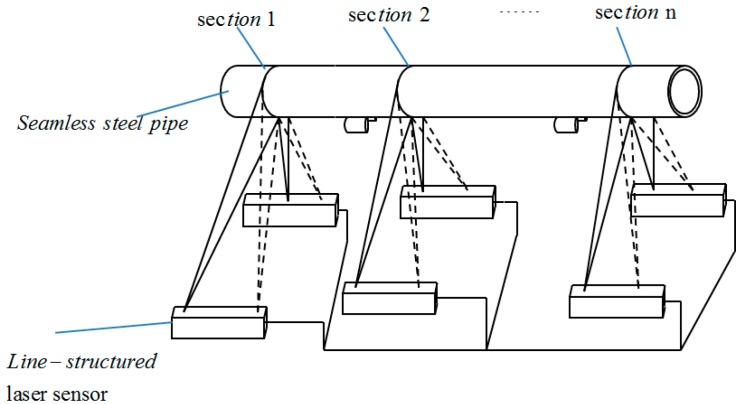
Sketch of the measurement system (with permission from [10]).

**Figure 4:**
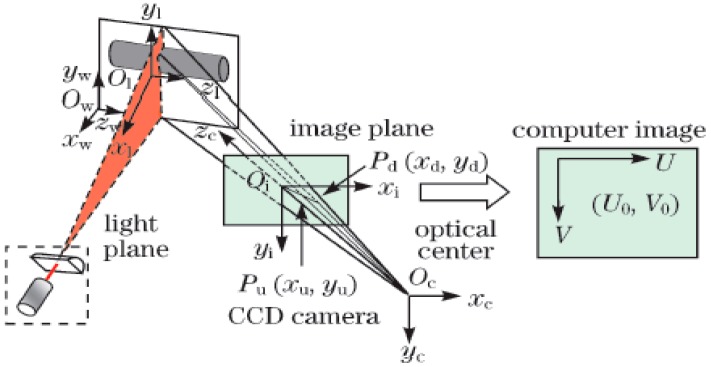
Mathematical model of the system (with permission from [11]).

Active measurement methods generally use one or more cameras and lasers to achieve accurate measurements of shaft diameters. In 2001, Sun *et al.* [10] used a pair of line-structured lasers and cameras to measure shaft diameters, as shown in Figure 3 [10]. The lights was projected onto the pipe, then captured by two cameras, and the diameter of a steel pipe could be determined by fitting the ellipse with the two developed arcs. It is difficult to ensure that the two arcs are on the same plane, so the accuracy of the method is limited. In 2010, Liu *et al.* [11] reported a method for measuring shaft diameters using a line-structured laser and a camera, as shown in Figure 4 [11]. The coordinates of the light stripe projected on the shaft were obtained using a novel gray scale barycenter extraction algorithm along the radial direction. The shaft diameter was then obtained by circle fitting using the generated coordinates. If the line-structured light is perpendicular to the measured shaft, the shaft diameter can be obtained by fitting a circle. Thus, this method is limited by the measurement environment.

In this study, a method for measuring shaft diameter is proposed using a line-structured laser and a camera. Based on the direction vector of the measured shaft axis, which is calibrated before the measurement, a virtual plane is established perpendicular to the axis and the image of a light stripe on the shaft is projected to the virtual plane. The shaft diameter is then determined from the projected image on the virtual plane. To improve the measuring precision, the center of the projected image was determined by fitting the projected image using a set of geometrical constraints.

This report is organized as follows: Section 2 calibrates the structured light system. Section 3 outlines the model of the proposed method. Section 4 reports the experimental results used to test the measuring accuracy and influences of several factors. Section 5 provides the study’s conclusions. 

## 2. Calibration of the Structure Light Measuring System

### 2.1. Calibration of Camera Parameters

**Figure 5:**
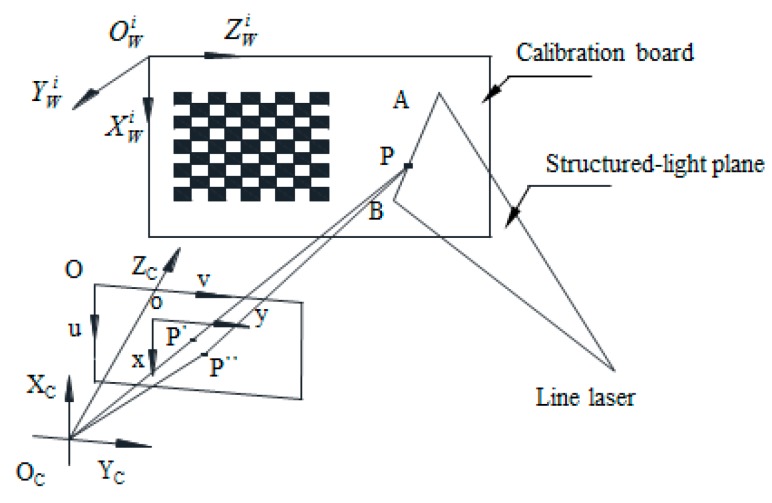
Calibration model of the line structured light.

The calibration of the measurement system employed world coordinate systems (O*^i^*_W_X*^i^*_W_Y*^i^*_W_Z*^i^*_W_), a camera coordinate system (O_C_X_C_Y_C_Z_C_), a image coordinate system (oxy), and a pixel coordinate system (Ouv), which were established as shown in Figure 5.

Based on pinhole projection and lens distortion models, the mapping from the world coordinates to the pixel coordinates can be expressed as reported by Zhang [12] and shown in Table 1, where R is the rotation matrix; T is translation vector; k_1_, k_2_, p_1_, and p_2_ are the coefficients of radial and tangential distortions; $A=\left[\begin{array}{ccc} \alpha & \gamma & u_{0}\\ 0 & \beta & v_{0}\\ 0 & 0 & 1 \end{array}\right]$ is the interior camera parameters; Z_W_ = 0 in the model. A nonlinear function can be established by minimizing the distance between the calculated pixel coordinates of the corner points in the calibration board and the actual pixel coordinates. The function was solved using the Levenberg-Marquardt algorithm [13].

**Table 1:** Coordinate transformation of camera model.

Transformation	Equations	
From world coordinates to camera coordinates	$\left[\begin{array}{c} X_{C}\\ Y_{C}\\ Z_{C} \end{array}\right]=R\left[\begin{array}{c} X_{W}\\ Y_{W}\\ 1 \end{array}\right]+T$	(1)
From camera coordinates to image coordinates	$\left[\begin{array}{c} x_{u}\\ y_{u} \end{array}\right]=\frac{1}{Z_{c}}\left[\frac{X_{c}}{Y_{c}}\right]$	(2)
Introduce the distortion model, where, r^2^=x^2^_d_+y^2^_d_,	$\left[\begin{array}{c} x_{u}\\ y_{u} \end{array}\right]=(1+k_{1}r^{2}+k_{2}r^{4})\left[\begin{array}{c} x_{d}\\ y_{d} \end{array}\right]+\left[\begin{array}{c} 2p_{1}x_{d}y_{d}+p_{2}\left(r^{2}+2x_{d}^{2}\right)\\ p_{1}\left(r^{2}+2y_{d}^{2}\right)+2p_{2}x_{d}y_{d} \end{array}\right]$	(3)
From image coordinates to pixel coordinates	$\left[\begin{array}{c} u\\ v\\ 1 \end{array}\right]=\left[\begin{array}{ccc} \alpha & \gamma & u_{0}\\ 0 & \beta & v_{0}\\ 0 & 0 & 1 \end{array}\right]\left[\begin{array}{c} x_{d}\\ y_{d}\\ 1 \end{array}\right]$	(4)

### 2.2. Calibration of Structured Light Parameters

The calibration model of the structured light is shown in Figure 5. Here, *AB* is the intersection line between the light plane and the calibration board, P is a point on *AB*. The pixel coordinate of P can be extracted as described by Steger [14], and camera coordinates of P are determined using Equations (1)–(4). Different intersection lines can be obtained by turning the board, so P*^i^_j_* is the *j*th point on the intersection line when the board is in the *i*th position.

Setting the equation of the light plane under O_C_X_C_Y_C_Z_C_:

(5) $$b_{1}X_{C}+b_{2}Y_{C}+b_{3}Z_{C}-1000=0$$

The parameters b_1_, b_2_, b_3_ can be determined by the objective function:

(6) $$\min\sum_{i=1}^{n}\sum_{j=1}^{k}\left\|b_{1}X_{Cj}^{i}+b_{2}Y_{Cj}^{i}+b_{3}Z_{Cj}^{i}-1000=0\right\|$$

where *n* is number of board turns, *k* is the number of sample points on the intersection line when the board is in the *i*th position, and (X*^i^*_C*j*_,Y*^i^*_C*j*_, Z*^i^*_C*j*_) are camera coordinates of P*^i^_j_*. According to the principle of least squares, the coefficients of Equation (5) can be solved as:

(7) $$\left[\begin{array}{c} b_{1}\\ b_{2}\\ b_{3} \end{array}\right]=1000\times {\left[\begin{array}{ccc} \sum_{i=1}^{n}\sum_{j=1}^{k}{{X^{i}}_{cj}}^{2} & \sum_{i=1}^{n}\sum_{j=1}^{k}{X^{i}}_{cj}{Y^{i}}_{cj} & \sum_{i=1}^{n}\sum_{j=1}^{k}{X^{i}}_{cj}{Z^{i}}_{cj}\\ \sum_{i=1}^{n}\sum_{j=1}^{k}{X^{i}}_{cj}{Y^{i}}_{cj} & \sum_{i=1}^{n}\sum_{j=1}^{k}{{Y^{i}}_{cj}}^{2} & \sum_{i=1}^{n}\sum_{j=1}^{k}{Y^{i}}_{cj}{Z^{i}}_{cj}\\ \sum_{i=1}^{n}\sum_{j=1}^{k}{X^{i}}_{cj}{Z^{i}}_{cj} & \sum_{i=1}^{n}\sum_{j=1}^{k}{Y^{i}}_{cj}{Z^{i}}_{cj} & \sum_{i=1}^{n}\sum_{j=1}^{k}{{Z^{i}}_{cj}}^{2} \end{array}\right]}^{-1}\left[\begin{array}{c} \sum_{i=1}^{n}\sum_{j=1}^{k}{X^{i}}_{cj}\\ \sum_{i=1}^{n}\sum_{j=1}^{k}{Y^{i}}_{cj}\\ \sum_{i=1}^{n}\sum_{j=1}^{k}{Z^{i}}_{cj} \end{array}\right]$$

Because the camera parameters and coordinates of the sample points have errors, the coefficients obtained by Equation (7) will be used as initial values for optimizing the light plane. The objective function of the optimized plane is established by the distances between the points and the plane:

(8) $$\min\sum_{i=1}^{n}\sum_{j=1}^{k}\frac{\left|b_{1}X_{Cj}^{i}+b_{2}Y_{Cj}^{i}\text{+}b_{3}Z_{Cj}^{i}-1000\right|}{\sqrt{{b_{1}}^{2}+{b_{2}}^{2}+{b_{3}}^{2}}}$$

The parameters of the plane can be obtained using the Levenberg-Marquardt algorithm. In the experiment, when the constant term of Equation (5) is 1, the parameters of the plane are small and the denominator of Equation (8) is around 0. To improve the optimization results, the constant term of Equation (5) is set at 1000 in the paper.

## 3. The Principle and Model of the Measurement

**Figure 6:**
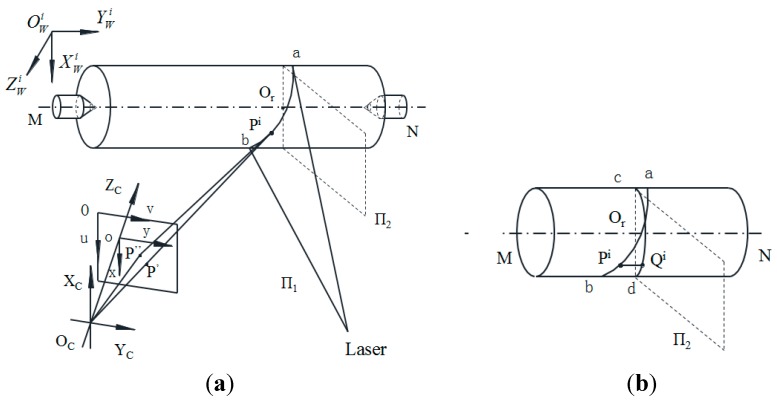
The measurement model of the shaft. (**a**) global view, (**b**) local view.

The model for measuring the shaft diameters is shown in Figure 6. A shaft is clamped at two centers, images of the shaft are captured by a CCD camera, п1 is the light plane, and *MN* is the axis of the shaft. The stripe *ab* is formed by projecting the light plane п1 onto the measured shaft. The pixel coordinates $P^{i}{\left(u_{p}^{i},v_{p}^{i}\right)}_{i=1,2,...,N}$ of *ab* stripe centers are extracted by Steger’s algorithm. The virtual plane п_2_ perpendicular to the measured shaft is created and the normal vector of п_2_ is the directional vector of the axis *MN*. Although the position of the axis changes slightly every time a shaft is clamped, the direction of the axis does not change. Thus, the equation of the virtual plane п_2_ can be obtained in O_C_X_C_Y_C_Z_C_ (see Appendix A for the details):

(9) $$b_{1}^{\prime }X_{C}+b_{2}^{\prime }Y_{C}+b_{3}^{\prime }Z_{C}-1000=0$$

The arc *cd* is formed by the virtual plane п_2_ intersecting the measured shaft.

Since the light plane п_1_ is not perpendicular to the shaft, the cross-section is an ellipse and the shaft diameter can be obtained by fitting the ellipse with stripe centers *P^i^*. The elliptic equation is:

(10) $$x^{2}+Axy+By^{2}+Cx+Dy+E=0$$

The parameters of Equation (10) can be obtained by the least squares method, and the length of the minor axis d is the measured shaft diameter:

(11) $$d=2\times \sqrt{\frac{2(ACD-BC^{2}-D^{2}+4BE-A^{2}E)}{\left(A^{2}-4B)(B+\sqrt{A^{2}+{\left(1-B\right)}^{2}}+1\right)}}$$

Since the virtual plane п_2_ is perpendicular to the shaft, the cross-section is a circle and the diameter can be obtained by fitting the circle with points *Q^i^*, which are *P^i^* projecting on the virtual plane п_2_. The circle equation is

(12) $$x^{2}+y^{2}+ax+by+c=0$$

The parameters of Equation (12) can be obtained by the least squares method, and the circle diameter d_1_ is the measured shaft diameter:

(13) $$d_{1}=2\times \sqrt{\frac{a^{2}+b^{2}}{4}-c^{2}}$$

Since the pixel coordinates of the extracted stripe centers have errors, from the numerical analysis, the measuring accuracy of the shaft diameter by circle fitting is better than by ellipse fitting (see Appendix B for the detail). 

The image coordinates $P_{\text{u}}^{i}={\left(x_{u}^{i},y_{u}^{i}\right)}_{i=1,2,3...N}^{T}$ of *P^i^* can be achieved using Equations (3) and (4), so the equations of *O_C_P^i^* are:

(14) $$\{\begin{array}{c} X_{P}={\text{x}}_{u}^{i}\cdot Z_{P}\\ Y_{P}=y_{u}^{i}\cdot Z_{P} \end{array}$$

The camera coordinates $P^{i}={\left(X_{P}^{i},Y_{P}^{i},Z_{P}^{i}\right)}_{i=1,2,3...N}$ can be obtained using Equations (5) and (14):

(15) $$\{\begin{array}{l} X_{P}^{i}=1000\times x_{u}^{i}/(b_{1}\cdot x_{u}^{i}+b_{2}\cdot y_{u}^{i}+b_{3})\\ Y_{P}^{i}=1000\times y_{u}^{i}/(b_{1}\cdot x_{u}^{i}+b_{2}\cdot y_{u}^{i}+b_{3})\\ Z_{P}^{i}=1000\times 1/(b_{1}\cdot x_{u}^{i}+b_{2}\cdot y_{u}^{i}+b_{3}) \end{array}$$

A set of lines *P^i^Q^i^* are made by passing through the extracted centers $P^{i}$ and parallel to the measured shaft axis, as shown in Figure 6b. Since the axis *MN* is perpendicular to the plane п_2_, the direction vectors of the lines are the normal vector of п_2_, $\left(b_{1}^{\prime },b_{2}^{\prime },b_{3}^{\prime }\right)$. Thus, the equations of lines *P^i^Q^i^* are:

(16) $$\{\begin{array}{c} \frac{x-X_{p}^{1}}{b_{1}^{\prime }}=\frac{y-Y_{p}^{1}}{b_{2}^{\prime }}=\frac{\text{z}-Z_{p}^{1}}{b_{3}^{\prime }}=t_{1}\\ \frac{x-X_{p}^{2}}{b_{1}^{\prime }}=\frac{y-Y_{p}^{2}}{b_{2}^{\prime }}=\frac{\text{z}-Z_{p}^{2}}{b_{3}^{\prime }}=t_{2}\\ \vdots \\ \frac{x-X_{p}^{N}}{b_{1}^{\prime }}=\frac{y-Y_{p}^{N}}{b_{2}^{\prime }}=\frac{\text{z}-Z_{p}^{N}}{b_{3}^{\prime }}=t_{N} \end{array}$$

Each line *P^i^Q^i^* has an intersection point *Q^i^* on the plane п_2_. The camera coordinates $Q^{i}{\left(x^{i},y^{i},z^{i}\right)}_{i=1,2,3...,N}$ of the intersection points are obtained using Equations (9) and (16), respectively.

**Figure 7:**
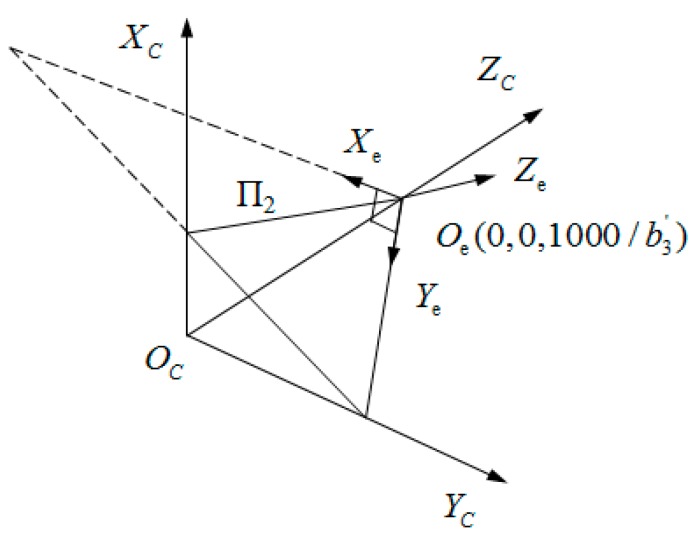
O_e_—X_e_Y_e_Z_e_ and O_C_—X_C_Y_C_Z_C_.

To facilitate the circle fitting, a coordinate system (O_e_—X_e_Y_e_Z_e_) is established on the plane п_2_, as shown in Figure 7. In O_e_—X_e_Y_e_Z_e_, X_e_Y_e_ plane is the plane п_2_, O_e_Z_e_ axis is perpendicular to the plane п_2_, and the origin of coordinate O_e_ is the intersection point of the plane п_2_ with O_C_Z_C_ axis. Additionally, camera coordinates of the origin of O_e_ $\left(0,0,1000/b_{3}^{\prime }\right)$ can be obtained by Equation (9). Thus, translations of the camera coordinate system are $\left(0,0,-1000/b_{3}^{\prime }\right)$ relative to O_e_—X_e_Y_e_Z_e_ and the camera coordinate Q^i^ can be changed into O_e_—X_e_Y_e_Z_e_ by Equation (17):

(17) $$\left[\begin{array}{c} x_{e}^{i}\\ y_{e}^{i}\\ z_{e}^{i} \end{array}\right]\text{=}\left[\begin{array}{ccc} \text{1} & 0 & 0\\ \text{0} & \text{cos}\theta & -\text{sin}\theta \\ \text{0} & \text{sin}\theta & \text{cos}\theta \end{array}\right]\left[\begin{array}{ccc} \text{cos}\psi & 0 & \text{sin}\psi \\ \text{0} & \text{1} & \text{0}\\ -\text{sin}\psi & 0 & \text{cos}\psi \end{array}\right]\left[\begin{array}{c} x^{i}\\ y^{i}\\ z^{i} \end{array}\right]+\left[\begin{array}{c} 0\\ 0\\ -1000/b_{3}^{\prime } \end{array}\right]$$

where, ψ is the rotation angle round O_C_Y_C_, θ is the rotation angle round O_C_X_C_, and $Q_{\text{e}}^{i}=(x_{e}^{i},y_{e}^{i},z_{e}^{i})$ are the coordinates of $Q^{i}$ in O_e_—X_e_Y_e_Z_e_. 

Normal vector $\bar{n}={\left(b_{1}^{\prime },b_{2}^{\prime },b_{3}^{\prime }\right)}^{T}$ of the plane п_2_ is converted by the coordinate rotation into ${\left(0,0,b_{3}^{\prime }/a\right)}^{T}$ in O_e_-X_e_Y_e_Z_e_, where, $a=\sqrt{{\left(b_{1}^{\prime }\right)}^{2}+{\left(b_{2}^{\prime }\right)}^{2}+{\left(b_{3}^{\prime }\right)}^{2}}$. Thus, $\bar{n}$ values before and after the conversion are substituted into Equation (18):

(18) $$\left[\begin{array}{c} 0\\ 0\\ b_{3}^{\prime }/a \end{array}\right]=\left[\begin{array}{ccc} \text{1} & 0 & 0\\ \text{0} & \text{cos}\theta & -\text{sin}\theta \\ \text{0} & \text{sin}\theta & \text{cos}\theta \end{array}\right]\left[\begin{array}{ccc} \text{cos}\psi & 0 & \text{sin}\psi \\ \text{0} & \text{1} & \text{0}\\ -\text{sin}\psi & 0 & \text{cos}\psi \end{array}\right]\left[\begin{array}{c} b_{1}^{\prime }\\ b_{2}^{\prime }\\ b_{3}^{\prime } \end{array}\right]$$

θ and ψ are obtained by Equation (18). In O_e_-X_e_Y_e_Z_e_, because points *Q^i^* are on п_2_, the coordinates *Q^i^* are $Q_{e}^{i}=(x_{e}^{i},y_{e}^{i},0)$.

**Figure 8:**
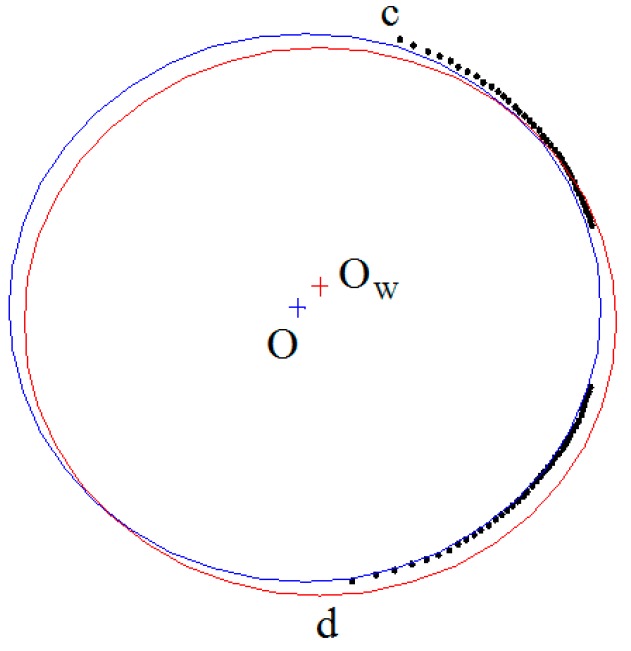
Fitting circle by limited data.

**Figure 9:**
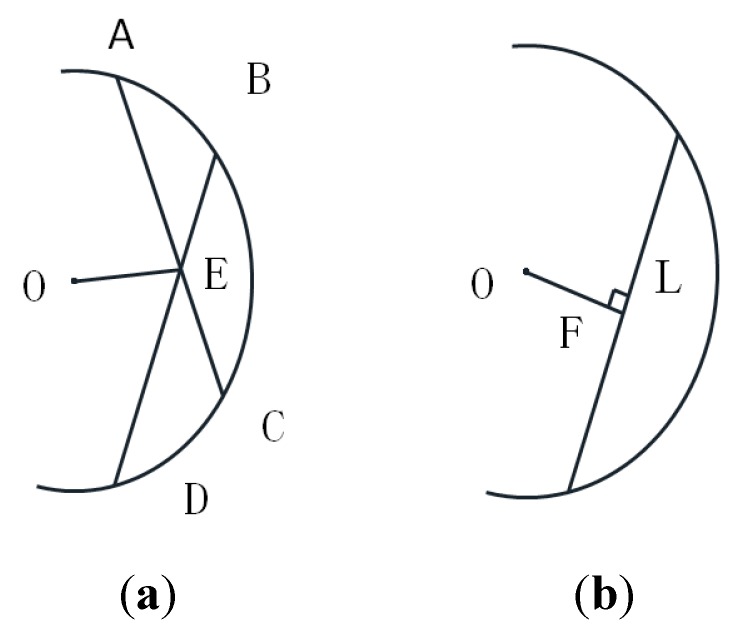
Geometrical features of a circle. (**a**) constraint condition1; (**b**) constraint condition2.

Since arc *cd* is at a side of the shaft and the coordinates of points *Q^i^* on *cd* have errors, the accuracy of the shaft diameter which is directly measured by fitting points on *cd* is poor, as shown in Figure 8. In Figure 8, the red circle is a cross-section formed by the virtual plane п_2_ intersecting with the shaft and O_w_ is the center of the red circle. The circle obtained by fitting the black points on *cd* is blue with its center at O. To improve the measuring accuracy, the center is determined by fitting black points under the geometrical constraints of arc *cd*, and the geometrical constraints for a circle center are shown in Figure 9. 

In Figure 9a, A, B, C, D are the fitting points in the circle and O is the center. Point E is the intersection point of *AC* and *BD*. From the geometrical feature of a circle, the following relation holds:

(19) $$AE*CE=BE*DE={\text{r}}^{2}-OE^{2}$$

where r is radius of the shaft. Setting the center coordinate as (x_0_,y_0_), the coordinates of the points A, B, C, D are (x_a_,y_a_), (x_b_,y_b_), (x_c_,y_c_), (x_d_,y_d_). The coordinate (x_e_,y_e_) of point E can be obtained from points A, B, C, D. Thus, the first objective function can be presented as:

(20) $$\min\sum_{i=1}^{n}D_{i}+DD_{i}$$

$$D_{i}=\sqrt{{\left(x_{ai}-x_{ei}\right)}^{2}+{\left(y_{ai}-y_{ei}\right)}^{2}}\times \sqrt{{\left(x_{ci}-x_{ei}\right)}^{2}+{\left(y_{ci}-y_{ei}\right)}^{2}}-r^{2}+{\left(x_{0}-x_{ei}\right)}^{2}+{\left(y_{0}-y_{ei}\right)}^{2}$$

$$DD_{i}=\sqrt{{\left(x_{bi}-x_{ei}\right)}^{2}+{\left(y_{bi}-y_{ei}\right)}^{2}}\times \sqrt{{\left(x_{di}-x_{ei}\right)}^{2}+{\left(y_{di}-y_{ei}\right)}^{2}}-r^{2}+{\left(x_{0}-x_{ei}\right)}^{2}+{\left(y_{0}-y_{ei}\right)}^{2}$$

where the value of r is estimated when optimizing Equation (20); n is number of the points.

As shown in Figure 9b, F is the midpoint of the chord L. Thus, line OF is perpendicular to the chord L and can be presented as:

(21) $$\text{min}\sum_{i=1}^{n}d_{i}+dd_{i}$$

$$d_{i}=(x_{0}-\frac{x_{ai}+x_{ci}}{2})(x_{ai}-x_{ci})+(y_{0}-\frac{y_{ai}+y_{ci}}{2})(y_{ai}-y_{ci})$$

$$dd_{i}=(x_{0}-\frac{x_{bi}+x_{di}}{2})(x_{bi}-x_{di})+(y_{0}-\frac{y_{bi}+y_{di}}{2})(y_{bi}-y_{di})$$

Therefore, the center coordinate can be determined by Equations (20) and (21):

(22) $$\min\sum_{i=1}^{n}D_{i}+DD_{\text{i}}+d_{i}+dd_{i}$$

Minimizing Equation (22) is a nonlinear minimization problem, which can be solved by the Levenberg-Marquardt algorithm. The initial value of the center is obtained by fitting the circle with the points in *cd*. 

Finally, the shaft diameter is obtained by the distance between the points in *cd* and the optimized center:

(23) $$d=2\times \frac{\sum_{i=1}^{n}\sqrt{{\left(x_{i}-X_{0}\right)}^{2}+{\left(y_{i}-Y_{0}\right)}^{2}}}{n}$$

where *d* is the diameter of shaft, (x_i_,y_i_) are coordinates of the points in *cd*, (X_0_,Y_0_) is the coordinate of the optimized center, and n is the number of fitting points.

## 4. Experiments and Analysis

**Figure 10:**
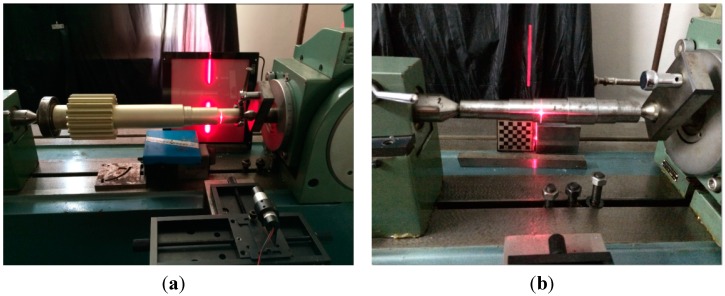
Experimental equipment for measuring shafts: (**a**) shaft 1; (**b**) shaft 2.

Experiments were conducted to assess the utility of the proposed method. The experimental equipment employed is shown in Figure 10 and the main parameters of the equipment are shown in Table 2. The interior parameters of the camera were calibrated by the method presented in Section 2. Two shafts were used in the experiments; shaft 1 was a four-segment shaft and the shaft surface’s reflection was reduced using a special process, as shown in Figure 10a. Shaft 2 was a seven-segment shaft, as shown in Figure 10b. The shafts’ diameters were measured using a micrometer with a resolution of 1 μm.

**Table 2:** Experimental equipment parameters.

Equipment	Mold NO.	Main Parameters
CCD camera	JAI CCD camera	Resolution: 1376 × 1024
Lens	M0814-MP	Focal length: 25 mm
Line projector	LH650-80-3	Wavelength: 650 nm
Mold plane CBC75mm-2.0		Precision of the grid: 1 μm

**Figure 11:**
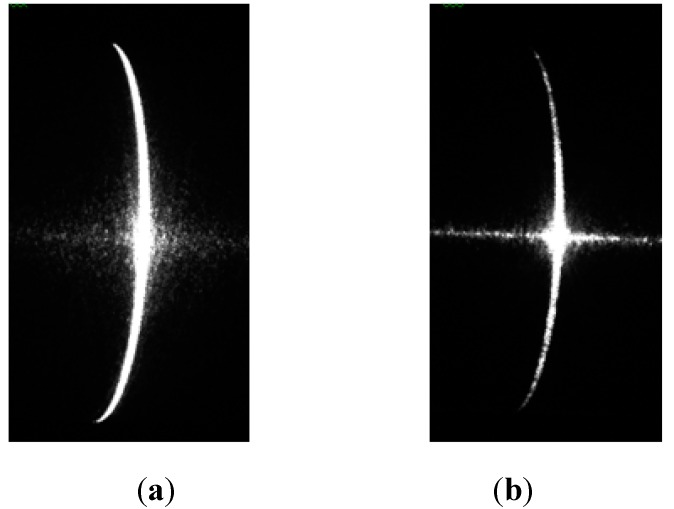
Images of stripes by camera: (**a**) shaft 1; (**b**) shaft 2.

First, the images of the shafts were captured using the camera, as shown in Figure 11. The diameters of the shafts were measured using the method described in Section 3. The measurement data are listed in Table 3 and Table 4, and the measurement error was less than 25 μm.

**Table 3:** Measurement results for shaft 1 (mm).

NO.	1	2	3	4
Measurement results	47.05	39.749	34.915	29.796
Known values	47.062	39.76	34.924	29.8
Errors	0.012	0.011	0.009	0.004

**Table 4:** Measurement results for shaft 2 (mm).

NO.	1	2	3	4	5	6	7
Measurement results	24.761	27.661	31.456	27.474	24.596	21.696	20.091
Known values	24.753	27.664	31.473	27.492	24.621	21.687	20.113
Errors	0.008	0.003	0.017	0.018	0.025	0.009	0.022

To compare the proposed method with the other methods, the diameters of the shafts were obtained by directly fitting the ellipse and circle. The data for these methods are shown in Table 5 and Table 6. The measuring accuracy of the present method was found to be better than that of the other two methods.

**Table 5:** Comparison of the measured diameters in shaft1 (mm).

NO.	D	Fitting Ellipse		Fitting Circle		Present Method	
		D1	Errors	D2	Errors	D3	Errors
1	47.062	46.992	0.07	47.034	0.028	47.05	0.012
2	39.76	39.659	0.101	39.698	0.062	39.749	0.011
3	34.924	34.773	0.151	34.827	0.097	34.915	0.009
4	29.8	29.741	0.059	29.73	0.07	29.796	0.004
Mean error			0.095		0.064		0.009

**Figure 12:**
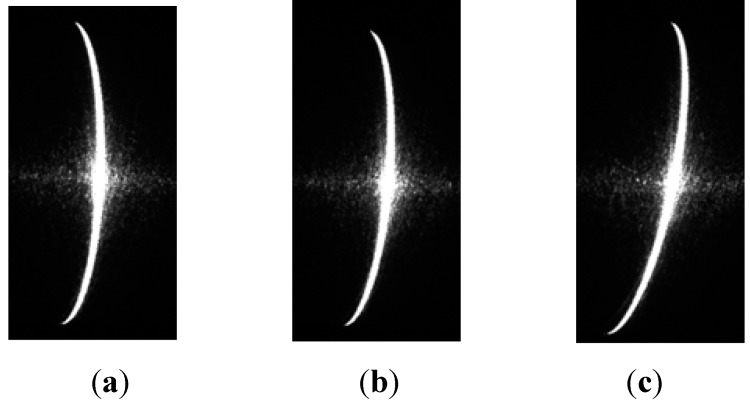
Images of stripes at three different angles.

To test the influence of the angle between the light plane and cross-section of the shaft on the proposed method, the shaft diameters were measured by light planes with three different light angles, as shown in Figure 12. Measurement results are listed in Table 7 and Table 8.

**Table 6:** Comparison of the measured diameters in shaft 2 (mm).

NO.	D	Fitting Ellipse		Fitting Circle		Present Method	
		D1	Errors	D2	Errors	D3	Errors
1	24.753	24.203	0.55	24.62	0.133	24.761	0.008
2	27.664	27.193	0.471	27.632	0.032	27.661	0.003
3	31.473	30.877	0.596	31.395	0.078	31.456	0.017
4	27.492	27.098	0.394	27.415	0.077	27.474	0.018
5	24.621	24.097	0.524	24.512	0.109	24.596	0.025
6	21.687	21.224	0.463	21.624	0.063	21.696	0.009
7	20.113	19.705	0.408	19.935	0.178	20.091	0.022
Mean error			0.487		0.096		0.015

From Table 7 and Table 8, the light angle appears to have very little effect on the measuring accuracy of the proposed method. 

**Table 7:** Errors for different angles in shaft 1 (mm).

NO.	1	2	3	4	Mean Error
a/Errors	0.012	0.011	0.009	0.004	0.009
b/Errors	0.014	0.012	0.012	0.012	0.013
c/Errors	0.01	0.016	0.013	0.008	0.012

**Table 8:** Errors for different angles in shaft 2 (mm).

NO.	1	2	3	4	5	6	7	Mean Error
a/Errors	0.008	0.003	0.017	0.018	0.025	0.009	0.022	0.015
b/Errors	0.005	0.007	0.004	0.016	0.026	0.013	0.018	0.013
c/Errors	0.008	0.013	0.025	0.017	0.028	0.009	0.022	0.017

**Figure 13:**
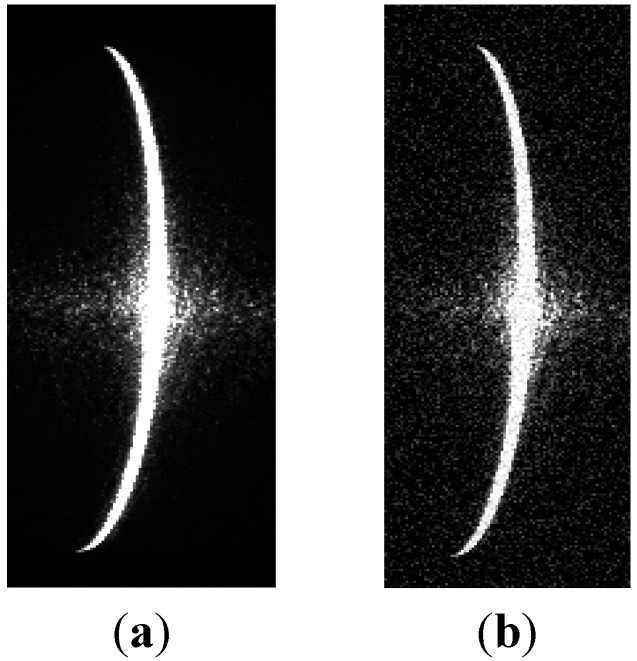
Images of stripe on shaft 1. (**a**) normal; (**b**) added noise.

**Figure 14:**
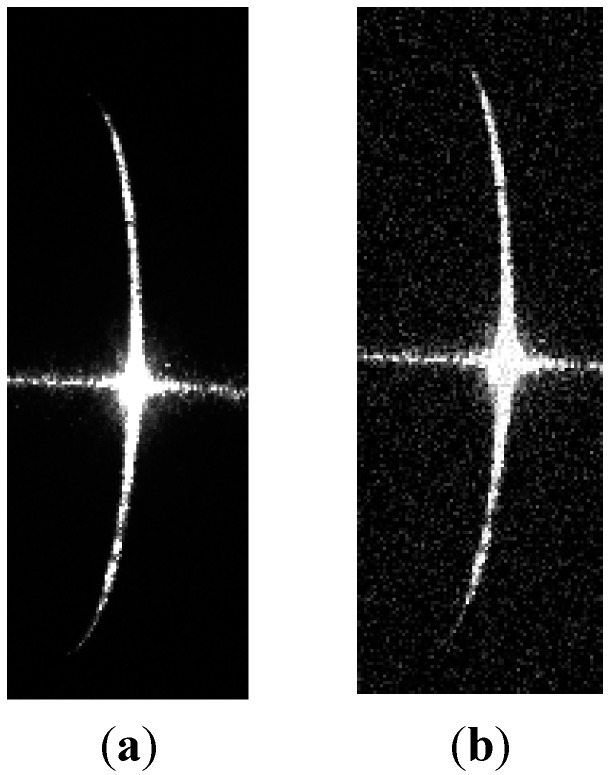
Images of stripe on shaft 2. (**a**) normal; (**b**) added noise.

In order to test the influence of noise on the proposed method, noise with a variance of 0.01 and a mean of zero was added to the stripe images of shaft 1 and shaft 2, as shown in Figure 13 and Figure 14. The center coordinates of the stripe images with the added noise were extracted by Steger’s algorithm [14]. The shaft diameters can be determined respectively by the ellipse, circle, and the proposed method fitting the center coordinates. The results are listed in Table 9 and Figure 10.

**Table 9:** Measurement results for shaft 1 (mm).

	D	Fitting Ellipse		Fitting Circle		Present Method	
		D1	Errors	D2	Errors	D3	Errors
Normal	34.924	34.924	0.151	34.827	0.097	34.915	0.012
Added noise	34.924	34.896	0.028	34.837	0.087	34.918	0.006
ΔD		0.123		0.01		0.003	

**Table 10:** Measurement results for shaft 2 (mm).

	D	Fitting Ellipse		Fitting Circle		Present Method	
		D1	Errors	D2	Errors	D3	Errors
Normal	31.473	30.877	0.596	31.395	0.078	31.456	0.017
Added noise	31.473	30.762	0.711	31.421	0.052	31.47	0.003
ΔD		0.115		0.026		0.014	

Here, the shaft diameters D were obtained by a micrometer, and ΔD is change of the shaft diameter measurements before and after the added noise. As seen in Table 9 and Table 10, the noise has little effect on the measuring accuracy of the proposed method.

## 5. Conclusions

A method for the measurement of shaft diameters is proposed which is based on a structured light vision measurement. A virtual plane is established perpendicular to the measured shaft axis and an equation for the virtual plane is determined using a calibrated model of structured light measurement. To improve the measurement accuracy, the center of the measured shaft can be obtained by fitting the projected light stripe image on the virtual plane under the geometrical constraints. Using specific experimental conditions, measurement errors of the method were less than 25 μm and the angle between the structured light plane and the measured shaft barely affected the measurement accuracy.

## Figures and Tables

**Figure A1:**
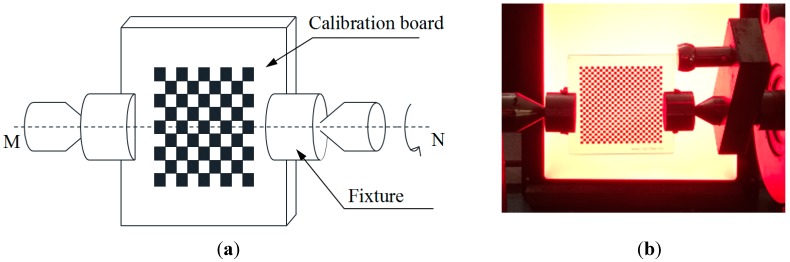
The model of the calibrating axis. (**a**) sketch map; (**b**) image of equipment.

## Appendix A

Since the virtual plane п_2_ is perpendicular to the measured shaft, the direction vector of the axis is the normal vector of п_2_. As shown in Figure A1, the calibration board is fixed by fixtures, and they are clamped by the two centers and the connecting line of the two centers is the axis of the shaft *MN*. Turning the calibration board, the images of the board can be captured by a camera and *MN* is the intersecting line of the calibration board planes.

Based on Equation (1), Equation (A1) can be obtained:

(A1) $$R_{j}^{-1}\cdot \left[\begin{array}{c} X_{C}-t_{1}^{j}\\ Y_{C}-t_{2}^{j}\\ Z_{C}-t_{3}^{j} \end{array}\right]=\left[\begin{array}{c} X_{W}^{\text{j}}\\ Y_{W}^{j}\\ Z_{W}^{j} \end{array}\right]$$

Set to $R_{j}^{-1}=D_{j}=\left[\begin{array}{ccc} d_{11}^{j} & d_{12}^{j} & d_{13}^{j}\\ d_{21}^{j} & d_{22}^{j} & d_{23}^{j}\\ d_{31}^{j} & d_{32}^{j} & d_{33}^{j} \end{array}\right]$, where, R*_j_* and t*_j_* are the rotation matrix and translation vector of the calibration target at the *j*th position and can be obtained by the method provided in Section 2, *j* = 1,2. Since world coordinate systems O*^j^*_W_X*^j^*_W_Y*^j^*_W_Z*^j^*_W_ are built on the calibration target plane, Z*^j^*_w_ = 0. The equation of the calibration target plane at the *j*th position is:

(A2) $$d_{31}^{j}X_{C}+d_{32}^{j}Y_{C}+d_{33}^{j}Z_{C}=d_{31}^{j}t_{1}^{j}+d_{32}^{j}t_{2}^{j}+d_{33}^{j}t_{3}^{j}$$

The normal vector of the plane is $S_{\text{j}}=\left(d_{31}^{j},d_{32}^{j},d_{33}^{j}\right)$. *MN* is the intersecting line of the two planes, so the direction vector of *MN* can be represented as:

(A3) $$S_{MN}=\left(d_{x},d_{y},d_{z}\right)=S_{1}\times S_{2}=\left({\text{d}}_{32}^{1}\cdot d_{33}^{2}-d_{33}^{1}\cdot d_{32}^{2},d_{33}^{1}\cdot d_{31}^{2}-d_{31}^{1}\cdot d_{33}^{2},d_{31}^{1}\cdot d_{32}^{2}-d_{32}^{1}\cdot d_{31}^{2}\right)$$

As shown in Figure 6, O_r_ is a point in п_2_ and set O_r_ is the intersection point of *MN* and п_1_, and the camera coordinate of O_r_ is O_r_ = (X_r_,Y_r_,Z_r_).

The equation of п_2_ in O_C_X_C_Y_C_Z_C_ is

(A4) $$d_{x}(X_{C}-X_{r})+d_{y}\left(Y_{C}-Y_{r}\right)+d_{z}\left(Z_{C}-Z_{r}\right)=0$$

Thus, equation parameters of the virtual plane can be obtained using Equations (9) and (A4):

(A5) $$\{\begin{array}{c} b_{1}^{\prime }=1000\times d_{x}/\left(d_{x}\cdot X_{\text{r}}+d_{y}\cdot Y_{r}+d_{z}\cdot Z_{r}\right)\\ b_{2}^{\prime }=1000\times d_{y}/\left(d_{x}\cdot X_{\text{r}}+d_{y}\cdot Y_{r}+d_{z}\cdot Z_{r}\right)\\ b_{3}^{\prime }=1000\times d_{z}/\left(d_{x}\cdot X_{\text{r}}+d_{y}\cdot Y_{r}+d_{z}\cdot Z_{r}\right) \end{array}$$

## Appendix B

$Q_{e}^{i}$ are the points for circle fitting, and the coordinates $Q_{\text{e}}^{i}={\left(x_{e}^{i},y_{e}^{i}\right)}_{i=1,2,3...,N}$ can be obtained by Equation (17). The coordinates $Q_{e}^{i}$ are substituted into Equation (12). Then Equation (12) is handled by the principle of least squares as:

(B1) $$A_{1}{\bar{x}}_{1}=b_{1}$$

where, $A_{1}=\left[\begin{array}{lll} \sum_{i=1}^{N}{\left(x_{e}^{i}\right)}^{2} & \sum_{i=1}^{N}x_{e}^{i}y_{e}^{i} & \sum_{i=1}^{N}x_{e}^{i}\\ \sum_{i=1}^{N}x_{e}^{i}y_{e}^{i} & \sum_{i=1}^{N}{\left(y_{e}^{i}\right)}^{2} & \sum_{i=1}^{N}y_{e}^{i}\\ \sum_{i=1}^{N}x_{e}^{i}\;\;\;\;\;\;\;\;\; & \;\;\sum_{i=1}^{N}y_{e}^{i}\;\;\;\;\;\;\;\;\;\;\;\;\;\;\; & \sum_{i=1}^{N}1\;\;\;\;\; \end{array}\right]$, $b_{1}=\left[\begin{array}{l} \sum_{\text{i}=1}^{N}{\left(x_{e}^{i}\right)}^{3}+x_{e}^{i}{\left(y_{e}^{i}\right)}^{2}\\ \sum_{i=1}^{N}{(x_{e}^{i}\text{)}}^{2}{\text{y}}_{e}^{i}+{\left(y_{e}^{i}\right)}^{3}\\ \sum_{i=1}^{N}{\left(x_{e}^{i}\right)}^{2}+{\left(y_{e}^{i}\right)}^{2}\;\;\;\;\;\;\;\; \end{array}\right]$, ${\bar{x}}_{1}=\left[\begin{array}{c} a\\ b\\ c \end{array}\right]$.

Using ${\bar{\text{x}}}_{1}$ obtained by Equation (B1), the measured shaft diameter can be obtained by Equation (13).

The coordinates of points for ellipse fitting are $P_{t}^{i}={\left(x_{t}^{i},y_{t}^{i}\right)}_{i=1,2,3...,N}$. To facilitate the ellipse fitting, a new coordinate system is established on the structured light plane п1, and the camera coordinates $P^{i}$ can be converted into $P_{t}^{i}={\left(x_{t}^{i},y_{t}^{i}\right)}_{i=1,2,3...,N}$ in the coordinate system. The coordinates $P_{t}^{i}$ are substituted into Equation (10). Then Equation (10) is handled by the principle of least squares as:

(B2) $$A_{2}{\bar{x}}_{2}=b_{2}$$

where, $A_{2}=\left[\begin{array}{ccccc} \sum_{i=1}^{N}{\left(x_{t}^{i}\right)}^{2}{\left(y_{t}^{i}\right)}^{2} & \sum_{i=1}^{N}x_{t}^{i}{\left(y_{t}^{i}\right)}^{3} & \sum_{i=1}^{N}{\left(x_{t}^{i}\right)}^{2}y_{t}^{i} & \sum_{i=1}^{N}x_{t}^{i}{\left(y_{t}^{i}\right)}^{2} & \sum_{i=1}^{N}x_{t}^{i}y_{t}^{i}\\ \sum_{i=1}^{N}x_{t}^{i}{\left(y_{t}^{i}\right)}^{3}\;\;\;\;\;\;\;\;\;\;\;\;\;\; & \sum_{i=1}^{N}{\left(y_{t}^{i}\right)}^{4}\;\;\;\;\;\;\;\;\;\;\; & \sum_{i=1}^{N}x_{t}^{i}{\left(y_{t}^{i}\right)}^{2} & \sum_{i=1}^{N}{\left(y_{t}^{i}\right)}^{3}\;\;\;\;\;\;\; & \sum_{i=1}^{N}{\left(y_{t}^{i}\right)}^{2}\\ \sum_{i=1}^{N}{\left(x_{t}^{i}\right)}^{2}y_{t}^{i}\;\;\;\;\;\;\;\;\;\;\;\;\;\;\; & \sum_{i=1}^{N}x_{t}^{i}{\left(y_{t}^{i}\right)}^{2} & \sum_{i=1}^{N}{\left(x_{t}^{i}\right)}^{2}\;\;\;\;\;\;\;\;\;\; & \sum_{i=1}^{N}x_{t}^{i}y_{t}^{i}\;\;\;\;\;\;\;\;\;\; & \sum_{i=1}^{N}x_{t}^{i}\\ \sum_{i=1}^{N}x_{t}^{i}{\left(y_{t}^{i}\right)}^{2}\;\;\;\;\;\;\;\;\;\;\;\;\;\; & \sum_{i=1}^{N}{\left(y_{t}^{i}\right)}^{3}\;\;\;\;\;\;\;\;\; & \sum_{i=1}^{N}x_{t}^{i}y_{t}^{i}\;\;\;\;\;\;\;\;\;\;\;\;\; & \sum_{i=1}^{N}{\left(y_{t}^{i}\right)}^{2}\;\;\;\;\;\;\; & \sum_{i=1}^{N}y_{t}^{i}\\ \sum_{i=1}^{N}x_{t}^{i}y_{t}^{i}\;\;\;\;\;\;\;\;\;\;\;\;\;\;\;\;\;\;\;\;\;\;\;\;\;\;\; & \sum_{i=1}^{N}{\left(y_{t}^{i}\right)}^{2}\;\;\;\;\;\;\;\;\;\; & \sum_{i=1}^{N}x_{t}^{i}\;\;\;\;\;\;\;\;\;\;\;\;\;\;\;\;\;\;\;\; & \sum_{i=1}^{N}y_{t}^{i}\;\;\;\;\;\;\;\;\;\;\;\;\;\;\;\;\;\;\; & \sum_{i=1}^{N}1 \end{array}\right]$, $b_{2}=\left[\begin{array}{l} \sum_{i=1}^{N}{\left(x_{t}^{i}\right)}^{3}y_{t}^{i}\\ \sum_{i=1}^{N}{\left(x_{t}^{i}\right)}^{2}{\left(y_{t}^{i}\right)}^{2}\\ \sum_{i=1}^{N}{\left(x_{t}^{i}\right)}^{3}\;\;\;\;\;\;\;\;\;\;\;\\ \sum_{i=1}^{N}{\left(x_{t}^{i}\right)}^{2}y_{t}^{i}\;\\ \sum_{i=1}^{N}{\left(x_{t}^{i}\right)}^{2}\;\;\;\;\;\;\;\;\;\; \end{array}\right]$, ${\bar{x}}_{2}=\left[\begin{array}{l} A\\ B\\ C\\ D\\ E \end{array}\right]$.

Using ${\bar{\text{x}}}_{2}$ obtained by Equation (B2), the measured shaft diameter can be obtained by Equation (11).

Since the matrixes (A_1_, A_2_) and the vectors (b_1_,b_2_) in Equations (B1) and (B2) consist of the coordinates of the light stripe centers, the matrixes and vectors are influenced by errors of the light stripe centers’ coordinates, which show that ${\bar{x}}_{1}$ and ${\bar{x}}_{2}$ are inaccurate. From *Numerical Analysis* (Seventh Edition) by Burden and Faires [15], the influence is analyzed as shown below.

Considering the errors of the light stripe centers’ coordinates, matrix A_1_ and vector b_1_ in Equation (B1) are expressed as:

$$A_{1}^{\prime }=A_{1}+\delta A_{1}\;;\;\;b_{1}^{\prime }=b_{1}+\delta b_{1}$$

where $\delta A_{1}$ and $\delta b_{1}$ are caused by errors of the light stripe centers’ coordinates. Thus, ${\bar{x}}_{1}$ in Equation (B1) becomes ${{\bar{x}}_{1}}^{\prime }={\bar{x}}_{1}+\delta {\bar{x}}_{1}$, and Equation (B1) can be expressed as:

(B3) $$\left(A_{1}+\delta A_{1})({\bar{x}}_{1}+\delta {\bar{x}}_{1})=(b_{1}+\delta b_{1}\right)$$

$$(A_{1}+\delta A_{1}){\bar{x}}_{1}+(A_{1}+\delta A_{1})\delta {\bar{x}}_{1}=b+\delta b_{1}$$

(B4) $$(A_{1}+\delta A_{1})\delta {\bar{x}}_{1}=\delta b_{1}-\delta A_{1}\cdot {\bar{x}}_{1}$$

Set $\left(A_{1}+\delta A_{1}\right)$ is an invertible matrix, and from Equation (B4) we get:

(B5) $$\delta {\bar{x}}_{1}={\left(A_{1}+\delta A_{1}\right)}^{-1}(\delta b_{1}-\delta A_{1}\cdot {\bar{x}}_{1})$$

In order to analyze the error of the fitting circle equation’s parameters, the norm of Equation (B5) is:

(B6) $$\left\|\delta x_{1}\right\|\leq \left\|{\left(A_{1}+\delta A_{1}\right)}^{-1}\right\|\cdot \left\|\delta b_{1}-\delta A_{1}\cdot {\bar{x}}_{1}\right\|$$

where $\left\|\delta x_{1}\right\|$ is error of the circle equation’s parameters ${\bar{x}}_{1}$ obtained by the circle fitting.

In the same way, the error of the equation’s parameters ${\bar{x}}_{2}$ for the ellipse fitting can be obtained by Equation (B2)

(B7) $$\left\|\delta x_{2}\right\|\leq \left\|{\left(A_{2}+\delta A_{2}\right)}^{-1}\right\|\cdot \left\|\delta b_{2}-\delta A_{2}\cdot {\bar{x}}_{2}\right\|$$

From Equations (B1) and (B2), ${\left(A_{1}+\delta A_{1}\right)}^{-1}$ and ${\left(A_{2}+\delta A_{2}\right)}^{-1}$ can be calculated by the light stripe centers’ coordinates.

When the measured shaft diameter is 24.753 mm, ${\left\|{\left(A_{1}+\delta A_{1}\right)}^{-1}\right\|}_{2}$ and ${\left\|{\left(A_{2}+\delta A_{2}\right)}^{-1}\right\|}_{2}$ can be calculated by the coordinates of the light stripe centers.

$${\left\|{\left(A_{1}+\delta A_{1}\right)}^{-1}\right\|}_{2}=27.04$$

$${\left\|{\left(A_{2}+\delta A_{2}\right)}^{-1}\right\|}_{2}=2454.06$$

So

(B8) $${\left\|\delta x_{1}\right\|}_{2}\leq 27.04{\left\|\delta b_{1}-\delta A_{1}\cdot {\bar{x}}_{1}\right\|}_{2}$$

(B9) $${\left\|\delta x_{2}\right\|}_{2}\leq 2454.06{\left\|\delta b_{2}-\delta A_{2}\cdot {\bar{x}}_{2}\right\|}_{2}$$

From Equations (B8) and (B9), ${\left\|\delta b_{2}-\delta A_{2}\cdot {\bar{x}}_{2}\right\|}_{2}$ must be very small if ${\left\|\delta x_{2}\right\|}_{2}\approx {\left\|\delta x_{1}\right\|}_{2}$. From Equation (B4), $\delta b_{2}-\delta A_{2}\cdot {\bar{x}}_{2}$ cannot be a zero vector. So, a very small ${\left\|\delta b_{2}-\delta A_{2}\cdot {\bar{x}}_{2}\right\|}_{2}$ requires that the absolute values of elements in the matrices $\delta A_{2}$ and the vector $\delta b_{2}$ must be very small. This shows that the errors of the light stripe centers’ coordinates for the ellipse fitting must be smaller than the ones for the circle fitting. On the other hand, if the coordinate errors of the light stripe centers for the circle fitting are the same as those for the ellipse fitting, the error of the circle equation parameters obtained by the circle fitting is smaller than the one of the ellipse equation parameters obtained by the ellipse fitting. From Equations (11) and (13), the measuring accuracy of the shaft diameter by circle fitting is better than by ellipse fitting. When the measured shaft diameter is 27.664 mm, ${\left\|{\left(A_{1}+\delta A_{1}\right)}^{-1}\right\|}_{2}=19.03$ and ${\left\|{\left(A_{2}+\delta A_{2}\right)}^{-1}\right\|}_{2}=1670.7$, the same results can be obtained.
